# Supplementary material for: The genome sequence of a mollusc, Azorinus chamasolen (da Costa, 1778)
Source: Wellcome Open Res. 2025 Jan 15;10:16. [Version 1] doi: 10.12688/wellcomeopenres.23457.1 (PMC12514443; doi:10.12688/wellcomeopenres.23457.1)
Supplement: Peer review history

# Reviewer report: Reviewer response for version 1

**DOI:** 10.21956/wellcomeopenres.25867.r117721

**Published:** 2025-10-10

**Authors:** Dongxiu Xue

**Affiliations:** 1 Qingdao Marine Science and Technology Center, Qingdao, China

This manuscript describes a high-quality, chromosome-level genome assembly of *Azorinus chamasolen* generated under the Darwin Tree of Life Project. The work is methodologically sound and provides valuable genomic resources for Solecurtidae.The manuscript is clear and well structured.

 However, The BUSCO completeness of 81.1% is somewhat lower than expected for chromosome-level molluscan assemblies. The authors should discuss potential biological or technical explanations.  

 In addition, Correct minor redundancy in the caption of Figure 2 (“The BlobToolKit snail plot shows…” repeated twice).

 I recommend **acceptance with minor revision**.

Are sufficient details of methods and materials provided to allow replication by others?

Yes

Is the rationale for creating the dataset(s) clearly described?

Yes

Are the datasets clearly presented in a useable and accessible format?

Yes

Are the protocols appropriate and is the work technically sound?

Yes

Reviewer Expertise:

Population genomics and genome evolution

I confirm that I have read this submission and believe that I have an appropriate level of expertise to confirm that it is of an acceptable scientific standard.

# Reviewer report: Reviewer response for version 1

**DOI:** 10.21956/wellcomeopenres.25867.r117726

**Published:** 2025-02-10

**Authors:** Nadège Guiglielmoni

**Affiliations:** 1 Universitat zu Koln, Cologne, North Rhine-Westphalia, Germany

This article presents a chromosome-level assembly for the mollusc *Azorinus chamasolen*. The haploid genome assembly spans 1.7 Gb with 19 chromosome candidates. The protocols for sequencing, assembly and evaluation are sound, but some useful information is missing.

 Hi-C preparation: I imagine that the 22% formaldehyde was not the final concentration of the buffer? For reproducibility, it would be better to indicate the final formaldehyde concentration and how long the sample was incubated in formaldehyde.

 Assembly curation: I would suggest to add a link for ASCC (I guess it would be https://github.com/sanger-tol/ascc).

 The evaluation part mentions Merqury but I do not see a k-mer plot. This would be necessary to understand whether the missing BUSCO orthologs are due to missing sequences in the assembly, or just absent in this species.

Are sufficient details of methods and materials provided to allow replication by others?

Partly

Is the rationale for creating the dataset(s) clearly described?

Partly

Are the datasets clearly presented in a useable and accessible format?

Yes

Are the protocols appropriate and is the work technically sound?

Yes

Reviewer Expertise:

Genome assembly of invertebrate animals

I confirm that I have read this submission and believe that I have an appropriate level of expertise to confirm that it is of an acceptable scientific standard, however I have significant reservations, as outlined above.

# Reviewer report: Reviewer response for version 1

**DOI:** 10.21956/wellcomeopenres.25867.r117475

**Published:** 2025-02-01

**Authors:** Kyle E McElroy

**Affiliations:** 1 Department of Ecology, Evolutionary, and Organismal Biology, Iowa State University, Ames, Iowa, USA

**Summary**

 The authors report details on the assembly of a chromosome-level genome assembly for a species of bivalve, *Azorinus chamasolen*. The assembly appears to be of high quality and completeness, as indicated by BUSCO and QV scores. I think the methods and results are clear and sufficiently detailed. I only have a few minor comments, with a larger question about how much biological context these reports should have.

**Major comments**

 While the focus of the Darwin Tree of Life Project is to “sequence all named eukaryotic species in the Atlantic Archipelago of Britain and Ireland”, I think at least a minimal amount of background on this animal would be nice. Is anything other than its name known? I have read and reviewed other Data Notes from Wellcome Open Research that were part of the Darwin Tree of Life Project and had background on the organism and motivation on sequencing its genome. Even if not much is known about this species, what is broadly known about the family or order? According to WORMS there are ~3000 species in Cardiida, making it among the most (if not *the *most) speciose order of bivalves. Only a few dozen have been sequenced at all, with just 17 chromosome-level reference genomes available on NBCI. I’d like to see some more biological context in this report.

 On a similar note, I think the title could be a bit more descriptive than “mollusc”. At the very least “bivalve mollusc (bivalve should also be a keyword, the species name doesn’t need to be a keyword).

**Minor comments**

 I appreciate the details of the Hi-C preparation. I have reviewed a few of these reports and for some reason those methods are often not nearly as detailed as the gDNA and RNA isolation. But the specific restriction enzyme used would still be a worthwhile detail to include. Was a particular enzyme needed as a parameter in analyzing the Hi-C data?

 What is “ *complete single alleles*” referring to regarding the Benchmark for Organelles?

Are sufficient details of methods and materials provided to allow replication by others?

Yes

Is the rationale for creating the dataset(s) clearly described?

Partly

Are the datasets clearly presented in a useable and accessible format?

Yes

Are the protocols appropriate and is the work technically sound?

Yes

Reviewer Expertise:

Evolutionary biology, genomics, genome evolution, bivalves, gene-family evolution, opsins, asexual reproduction, transposable elements

I confirm that I have read this submission and believe that I have an appropriate level of expertise to confirm that it is of an acceptable scientific standard, however I have significant reservations, as outlined above.
